# Nutritional Laboratory Markers in Malnutrition

**DOI:** 10.3390/jcm8060775

**Published:** 2019-05-31

**Authors:** Ulrich Keller

**Affiliations:** FMH Endocrinology-Diabetology, Fichtlirain 33, CH-4105 Biel-Benken, Basel, Switzerland; ulrich.keller@unibas.ch

**Keywords:** malnutrition, inflammation, nutritional assessment, biomarkers, albumin, prealbumin, IGF-1, elderly, prognostic marker

## Abstract

Serum visceral proteins such as albumin and prealbumin have traditionally been used as markers of the nutritional status of patients. Prealbumin is nowadays often preferred over albumin due to its shorter half live, reflecting more rapid changes of the nutritional state. However, recent focus has been on an appropriate nutrition-focused physical examination and on the patient’s history for diagnosing malnutrition, and the role of inflammation as a risk factor for malnutrition has been more and more recognized. Inflammatory signals are potent inhibitors of visceral protein synthesis, and the use of these proteins as biomarkers of the nutritional status has been debated since they are strongly influenced by inflammation and less so by protein energy stores. The current consensus is that laboratory markers could be used as a complement to a thorough physical examination. Other markers of the nutritional status such as urinary creatinine or 3-methylhistidine as indicators of muscle protein breakdown have not found widespread use. Serum IGF-1 is less influenced by inflammation and falls during malnutrition. However, its concentration changes are not sufficiently specific to be useful clinically as a marker of malnutrition, and serum IGF-1 has less been used in clinical trials. Nevertheless, biomarkers of malnutrition such as prealbumin may be of interest as easily measurable predictors of the prognosis for surgical outcomes and of mortality in severe illnesses.

## 1. Introduction

Malnutrition has a substantial clinical and socioeconomic significance; it increases rates of complications in hospitalized patients and healthcare-associated costs. Its prevalence has been estimated in hospitals of Western countries to be 30–50% and in long-term care facilities up to 85% depending on the definition and the type of population studied [1,2,3].

One of the problems of diagnosing malnutrition is the lack of a unified definition and of standard methods for screening and diagnosis. 

Malnutrition results from a mismatch of nutritional requirements with intake. In many malnourished patients, there is an associated disease-related inflammation, resulting in a complex interplay between the two. Inflammation influences both, requirements and intake. It promotes malnutrition and adverse outcomes by provoking anorexia and by altering metabolism with elevation of resting energy expenditure and by increasing muscle catabolism [4].

The appreciation that inflammation plays a major role in the pathophysiology of malnutrition is often lacking, and many clinicians assume that weight loss is the single most important criterion for a malnourished state. This has led to frequent underrecognition of the condition [5]. 

The purpose of this review is to review the published literature on the role of laboratory biomarkers as a tool to diagnose malnutrition, to assess nutritional risk and to monitor nutritional intervention.

## 2. Role of Biomarkers in Published Screening Tools to Assess the Risk of Malnutrition

About half of all published risk scores of malnutrition use serum laboratory markers such as visceral proteins, and others do not (Table 1).

Results of these screening tests vary considerably, as shown in a Greek study in elderly subjects [2]. These authors found the highest validity coefficient for MUST but a lower specificity for NRS 2000 which has frequently been used.

A consensus committee of the European and US nutritional societies (ESPEN and ASPEN) proposed three sub-definitions of malnutrition. “Starvation-related malnutrition” is present when there is chronic starvation without inflammation; “chronic disease-related malnutrition” is defined as a condition when inflammation is chronic and of mild to moderate degree, and “acute disease or injury-related malnutrition” occurs when inflammation is acute and of severe degree [21]. The criteria for diagnosis of these sub-definitions were energy intake, weight loss, body fat, muscle mass, fluid accumulation and grip strength but no biomarkers [22]. Among ancillary parameters, serum visceral proteins were mentioned, but since they would rather reflect the state of inflammation it was proposed to use them with caution to diagnose malnutrition. 

A meta-regression published recently [23] assessed the role of biomarkers in describing the severity of malnutrition according to established and validated nutritional assessment tools. A total of 111 studies (observational and cohort studies; randomized controlled trials were not available) were included, representing 52,911 participants from various clinical settings. The BMI (*p* < 0.001) and concentrations of albumin (*p* < 0.001), hemoglobin (*p* < 0.001), total cholesterol (*p* < 0.001), prealbumin (*p* < 0.001) and total protein (*p* < 0.05) among subjects at high risk of malnutrition assessed by MNA were significantly lower than those with low risk. Similar results were observed for malnutrition identified by SGA and NRS 2002. When patients with acute illnesses were included, the predictive value of albumin and prealbumin was distinctly reduced, confirming the conclusion that they are more markers of inflammation than of malnutrition. The authors concluded that BMI, hemoglobin, and total cholesterol were useful markers of malnutrition in older adults. 

## 3. Serum Visceral Proteins as Biomarkers of the Nutritional Status

Visceral proteins are mostly synthesized in the liver. Poor protein and energy intake, impaired liver synthetic function as well as inflammatory status result in low circulating levels of visceral proteins. During inflammatory states and increased production of acute-phase proteins the liver reprioritizes protein synthesis, and lowers as a mirror visceral protein synthesis to a degree which correlates with the severity of the injury.

### 3.1. Serum Albumin

Albumin is the most abundant protein in human serum. It has been used for decades as an indicator of malnutrition in patients in clinically stable conditions (review and meta-analysis [24]). Serum albumin concentrations decrease with increasing age by approx. by 0.1 g/L per year; however, age itself is not a cause of distinct hypoalbuminemia. 

There is a clear relationship between serum albumin concentrations and all-cause mortality in elderly subjects [25]. In patients with a hip fracture, albumin levels below 35 g/L were associated with higher rates of post-operative complications such as sepsis and higher overall mortality. Significant loss of muscle mass has been observed in elderly people with low albumin levels. Inflammatory states and in particular, high concentrations of the cytokines IL-6 and TNF-alpha, were two of the main factors causing low levels of serum albumin [24]. Systemic inflammation not only reduces albumin synthesis but increases its degradation and promotes its transcapillary leakage.

Other studies also found this protein to be a good predictor of surgical outcome [26,27]. Compared to nine other risk variables, serum albumin was the strongest predictor. These findings were confirmed in a later study [26], but whether hypoalbuminemia was due to undernutrition or advanced disease was not clarified in these trials.

When serum albumin as a biomarker for the differential diagnosis of unexplained weight loss (involuntary weight loss of more than 5 kg in the previous 6 months) was included in a study from Spain in 306 referred patients, a little more than one-third were ultimately diagnosed with a malignancy. Multivariate analysis found the strongest predictors of a neoplasm were age >80 years, white blood cell count > 12,000/mm^3^ and serum albumin < 3.5 g/dL [28].

Albumin has been criticized as a player in nutritional assessment due to its lack of specificity and long half-life (approximately 20 days) [29]. Serum albumin concentrations not only decrease during decreased synthesis due to inflammatory cytokines as mentioned above or to hepatic insufficiency, they may also decrease following renal losses in nephrotic syndrome and to losses via the GI tract in protein-losing enteropathies [30].

### 3.2. Serum Prealbumin 

Prealbumin, also named transthyretin, is a transport protein for thyroid hormone and is synthesized by the liver and partly catabolized by the kidneys. Serum prealbumin concentrations less than 10 mg/dL are associated with malnutrition [31].

The use of prealbumin has been advocated as a nutritional marker, particularly during refeeding and in the elderly [32]. The main advantage of prealbumin compared to albumin is its shorter half-life (two to three days) (Table 2), making it a more favorable marker of acute changes of the nutritional state. In addition, prealbumin was not influenced by intestinal protein losses in patients with protein-losing enteropathy [30].

Prealbumin levels may be increased in the setting of renal dysfunction, corticosteroid therapy or dehydration, whereas they can be decreased during physiological stress, infection, liver dysfunction, and over-hydration [34].

An algorithm that uses prealbumin has recently been proposed as a practical guide to help the clinician to stratify general medical and intensive care patients by risk of complications and outcome [34]. Prealbumin screening should only performed when an acute inflammatory state (CRP > 15 mg/L) was excluded. A prealbumin level of < 0.11 g/L was associated with increased mortality and length of stay, and an increase by less than 0.04 g/L per week indicated failure of nutritional therapy.

An increase in the C-reactive protein/prealbumin ratio in medical intensive care unit patients has been associated with mortality [35], and a low C-reactive protein/prealbumin ratio in surgical patients predicted the successful closure of gastrointestinal fistulas [36]. Routine measurement of prealbumin has been advocated to be a useful nutritional and prognostic indicator in non-ICU patients without inflammation [34].

Several publications reported a role for prealbumin in predicting prognosis (mostly survival) in various clinical conditions such as gastric cancer [37], lung cancer [38] and cardiovascular diseases [39].

### 3.3. Albumin and Prealbumin in Starved and Otherwise Healthy Malnourished Subjects

A systematic review assessed the role of albumin and prealbumin in otherwise healthy subjects who were severely nutrient- deprived due to poor access to food or unwillingness to eat, mostly due to anorexia nervosa [40]. The study showed that serum albumin and prealbumin levels were maintained even in the presence of distinct weight loss, and they were lowered only during extreme starvation (BMI < 11 kg/m^2^). The authors concluded that serum visceral proteins are not predictive of nutritional deprivation and should not be used to guide nutritional therapy in this group of patients.

### 3.4. Transferrin

This acute-phase reactant is a transport protein for iron. It has a relatively long half-life (approx. 10 days), and has also been used as a marker of the nutritional status [41] It is influenced by other factors including iron status, liver disease and inflammatory state. Like prealbumin, transferrin levels increase with renal failure [42]. Some authors found transferrin measurements useful for nutritional assessment [43], other did not [44].

During iron-deficiency the levels of transferrin are elevated whereas they are decreased in iron-overload states. Serum transferrin increased in parallel to prealbumin during nutritional intervention in critically ill children [45]. Serum levels decrease in the setting of severe malnutrition, but this marker has been found to be unreliable in the assessment of mild malnutrition and of fat-free mass in a group of elderly Italian patients [46].

### 3.5. Retinol-Binding Protein (RBP)

This is a low molecular weight protein with the physiological role to transport retinol from the liver to target organs. It represents the visceral protein with the shortest half-life (approx. 12 h) [33]. According to a review [47] it provides similar responses to energy intake to prealbumin, but it is more difficult to measure than the latter and it is influenced by the vitamin A status. For these reasons there RBP measurements have not found widespread application.

## 4. Laboratory Markers of Malnutrition Other Than Visceral Proteins

### 4.1. Urinary Creatinine

Creatinine is the end product of creatine which consists of 3 amino acids and is mainly present in muscle. Provided that renal function is intact its excretion reflects creatinine production which in turn is a mirror of skeletal muscle turnover. Each mmol of creatinine in urine is derived from 1.9 kg skeletal muscle [47]. The disadvantages are that it is slowly responding to changes of the nutritional status and that it depends on renal function and requires 24 h urine collections.

### 4.2. Urinary 3-Methylhistidine

3-methylhistidine is a component of muscle fibers and is not reutilized by the body. Its urinary excretion reflects the amount of fat-free mass and it can be used as a measure of the rate of muscle protein breakdown. It is less dependent on renal function than creatinine; it is often expressed per mmol of urinary creatinine [47]. Both assay of urinary creatinine and 3-methylhistidine have not found widespread use mainly due to the fact that urinary collections are often cumbersome, their excretion may increase after meat intake and they show a relatively poor sensitivity to monitor changes of body protein stores.

### 4.3. Serum Cholesterol

As can be seen in Table 1, some nutritional screening tools used total serum cholesterol as parameter of malnutrition. Serum cholesterol concentrations show a U-shaped relationship with mortality, and low levels have been associated with increased mortality [48]. However, sensitivity and specificity to monitor malnutrition are low.

### 4.4. Delayed Hypersensitivity and Blood Lymphocyte Count

The local inflammatory response to a s.c. injection of an antigen is impaired during severe malnutrition. At the same time, maturation of lymphocytes may be reduced in malnourished patients so that total circulating lymphocyte concentration falls to less than 1500/mm^3^ (reference range 2000–3500) [47].

These abnormalities can be taken as supporting evidence for protein-energy malnutrition, however, they are not specific and insensitive, and concomitant diseases and a severe stress reaction may also have an effect. Both markers respond slowly to correction of the nutritional status. These reasons limit their use as diagnostic tools for malnutrition.

### 4.5. Serum Insulin-Like Growth Factor 1 (IGF-1)

IGF-1 (formerly called Somatomedin C) is a ubiquitous growth factor, and the circulating form is mainly produced by the liver. Pituitary growth hormone stimulates its release. Its serum half-life is short (approx. 24 h), and it is largely bound in plasma to binding proteins (mainly IGF BP 3). Fasting lowers plasma IGF-1 levels more than 4 fold and IGF-1 concentrations increases during nutritional repletion. A correlation between energy intake (and less so of protein intake) and plasma IGF-1 concentrations has been reported [49]. IGF-1 levels were a reliable index of protein-energy undernutrition in elderly patients in the recovery period after surgery for hip fracture; however, according to this trial, this marker was also influenced by inflammation [50]. In contrast, IGF-1 levels were not clearly influenced by inflammation in other groups of surgical patients [51,52]. IGF-1 concentrations are altered by liver disease, renal impairment and severe trauma such as burns [47]. Nevertheless, IGF-1 performed better during nutritional rehabilitation to monitor protein and energy status than albumin or transferrin [53]. Drawback of IGF-1 measurements is the fact that their serum concentrations are influenced by other factors such as the acute-phase response. More recently, there has been interest in free IGF-I which holds promise as a nutritional marker (review in [54]. In spite of the earlier positive reports, IFG-1 measurements have not been advocated in more recent publications.

### 4.6. Serum Leptin

Decreased serum leptin concentrations combined with elevated prothrombin time has been reported in malnourished hospitalized patients with end-stage liver disease [55].

### 4.7. Serum Nesfatin-1

Nesfatin-1 is an anorexigenic molecule and seems to play a role in appetite regulation and energy homeostasis. Serum nesfatin-1 concentrations have been shown to be increased in chronically malnourished but otherwise healthy children [56].

### 4.8. Serum Zinc

Zinc is the most abundant trace element in man beside iron; it is present in all body tissues and fluids and is an essential component of many enzymes. Zinc deficiency has been associated with impaired taste and smell, reduced immunity and increased risk of pneumonia [57]. In cases of severe zinc deficiency, skin lesions, anemia, diarrhea, anorexia, decreased lymphocyte function, impaired visual function and mental retardation may be observed. Several psychological functions were impaired in elderly subjects with zinc deficiency [58].

Zinc deficiency is due to low intake of zinc-containing foods such as meat and to decreased absorption caused by intestinal malabsorption [57]. According to a large sample of the TromsØ study, the risk of zinc deficiency was increased 3 fold in subjects at high risk of malnutrition, particularly in men [59]. Assessment of the zinc status carries the problems that only a small fraction of body zinc is circulating, and most serum zinc is bound to albumin. Therefore, albumin deficiency makes interpretation of serum zinc levels difficult. In spite of the widespread functions of zinc in the body and the potential importance replacing zinc in subjects with zinc deficiency, there is little high-quality evidence of the therapeutic benefit of zinc replacement in adult subjects. A randomized controlled trial in children with protein-energy malnutrition and zinc deficiency showed benefits of zinc replacement [60]. It is likely that zinc deficiency in subjects at risk of malnutrition remains often unrecognized.

### 4.9. Other Essential Micronutrients (Trace Elements and Vitamins)

Laboratory assessment of other trace elements such as iron is not specifically mentioned as part of current nutritional screening tools. This does not mean that in cases with clinical suspicion of micronutrient deficiency this should not be performed. The same can be stated for laboratory screening for vitamin deficiencies, in particular, those of vitamins A, B1, B6, B12, D, and folate.

## 5. Biomarkers of Nutritional Risk in Some Specific Clinical Conditions

### 5.1. Geriatric Patients

**Dementia:** Eating and swallowing problems increase the risk of malnutrition. According to a study from a memory clinic in the Netherlands, about 14% of community-dwelling subjects with newly diagnosed dementia were at risk of malnutrition [61]. These authors pointed out that it is important to detect malnutrition in dementia as early as possible.

In patients with dementia, the nutritional history may only be obtainable by asking family caregivers about appetite and weight change of patients. However, it is unknown whether nutritional information provided by family caregivers are reliable, and therefore biomarkers of malnutrition would be of particular interest.

A recent study compared biochemical blood markers among patients with Alzheimer’s disease (AD), dementia with Lewy bodies (DLB), and frontotemporal lobar degeneration (FTLD) [62]. A total of 339 dementia outpatients and their family caregivers participated. Low serum albumin was 7.2 times more prevalent among patients with DLB and 10.1 times more prevalent among those with FTLD than among those with AD, with adjustment for age. The levels of biochemical markers were not significantly correlated with cognitive function. These authors proposed that a multidimensional approach including serological biomarkers such as albumin are needed to assess malnutrition in patients with dementia.

**Sarcopenia in the elderly:** A clinical investigation performed in elderly persons supported the view that prealbumin levels are useful surrogate indicators of lean body mass (LBM). Compared to serum albumin and RBP, prealbumin showed the highest positive correlation with LBM [46]. In order to improve its predictive potency for sarcopenia the reference values for prealbumin should be adapted to the corresponding age and sex [32]. Ingenbleek proposed that routine screening for protein malnutrition using prealbumin should be performed in elderly subjects [63].

### 5.2. Chronic Kidney Disease

A position paper from the International Society of Renal Nutrition and Metabolism (ISRNM) stated that serum biomarkers played a particular role in diagnosing malnutrition in patients with kidney failure [64]. Protein-energy wasting can be observed in chronic and in acute kidney disease, and protein-energy wasting is diagnosed according to this publication when low serum biomarkers (albumin, prealbumin, or cholesterol), reduced body mass and reduced muscle mass are present.

Serum prealbumin levels were positively correlated with body cell mass in pre-dialytic kidney patients [65]. Serum biomarkers were part of a new nutritional risk index for predicting mortality in Japanese hemodialysis patients [66]. Cox proportional hazard models indicated that in addition to low BMI, low albumin, low creatinine, and low serum cholesterol predicted independently and significantly mortality within one year.

## 6. Conclusions

The role played by serum biomarkers in diagnosing or monitoring malnutrition is controversial, particularly in more recent reports. This is explained by their relatively low specificity and by the fact that underlying diseases such as inflammation exert a major influence, particularly on serum visceral proteins. In addition, the role of biomarkers to guide nutritional therapy has not been studied in large randomized controlled trials. A recent randomized controlled multicenter trial in hospitalized patients with malnutrition might fill this gap in the near future [67]. Nevertheless, biomarkers such as prealbumin are valid prognostic indicators of disease outcome and of mortality in patients at risk of malnutrition.

## Figures and Tables

**Table 1:** Anthropometric parameters and biomarkers in various nutritional assessment and screening tools (adapted from [3] with an update, in chronological order of publication.

Nutritional Assessment and Screening Tool	Anthropometric Parameters and History	Biomarkers
Prognostic Nutritional Index [6]	Triceps skin fold	Albumin, transferrin, skin sensitivity
Prognostic Inflammatory and Nutritional Index [7]	None	Albumin, prealbumin, C-reactive protein, α1-acid glycoprotein
Subjective Global Assessment (SGA) [8]	Weight history, diet history, primary diagnosis, stress level, physical symptoms (s.c. fat, muscle wasting, edema), functional capacity, gastrointestinal symptoms	None
Birmingham Nutrition Risk Score [9]	Weight loss, BMI, appetite, ability to eat, stress factor, (severity of diagnosis)	None
Nutrition Risk Classification [10]	Weight loss, percentage ideal body weight, dietary intake, gastrointestinal function	None
Mini Nutritional Assessment (MNA; [11]	Weight data, height, mid-arm circumference, calf circumference, diet history, appetite, feeding mode	Albumin, prealbumin, cholesterol, lymphocyte count
Malnutrition Screening Tool [12]	Appetite, unintentional weight loss	None
Simple Screening Tool [13]	Body mass index (BMI), percentage weight loss	Albumin
Full nutritional assessment [14]	BMI, information on unintended weight loss, triceps skinfold thickness, mid-arm muscle circumference	Serum albumin, prealbumin, and total lymphocyte count
Malnutrition Universal Screening Tool (MUST) [15]	BMI, change in weight, presence of acute disease	None
Nutritional Risk Screening (NRS) 2002 [1]	Weight loss, BMI, food intake, diagnosis (severity)	None
Short Nutrition Assessment Questionnaire [16]	Recent weight history, appetite, use of oral supplement or tube feeding	None
Controlling nutritional status (CONUT) [17]	None	Serum albumin, total cholesterol and total lymphocyte count
Maastricht Index [18]	Percentage ideal body weight	Albumin, prealbumin, lymphocyte count
Nutritional Risk Index [19]	Present and usual body weight	Albumin
Elderly Nutritional Indicators for Geriatric Malnutrition Assessment (ENIGMA) [20]	Nutritional history	Albumin, hemoglobin, total cholesterol and lymphocyte count

**Table 2:** Characteristics of serum visceral proteins used as nutritional markers.

Protein	Molecular Weight	Half-Life	Reference Range
Albumin	65,000	20 days	3.30 to 4.80 g per dL
Transferrin	76,000	10 days	0.16 to 0.36 g per dL
Prealbumin	54,980	2 days	16 to 35 mg per dL
Retinol-binding protein	21,000	½ day	3–6 mg/dL

Table adapted from Spiekerman AM [33].
